# Colonization of *Solanum melongena* and *Vitis vinifera* Plants by *Botrytis cinerea* Is Strongly Reduced by the Exogenous Application of Tomato Systemin

**DOI:** 10.3390/jof7010015

**Published:** 2020-12-29

**Authors:** Donata Molisso, Mariangela Coppola, Anna Maria Aprile, Concetta Avitabile, Roberto Natale, Alessandra Romanelli, Pasquale Chiaiese, Rosa Rao

**Affiliations:** 1 Department of Agricultural Sciences, University of Naples Federico II, Via Università 100, Portici, 80055 Naples, Italy; donata.molisso@unina.it (D.M.); mariangela.coppola@unina.it (M.C.); annamaria.aprile@unina.it (A.M.A.); roberto.natale@unina.it (R.N.); 2 CNR-IBB, Via Mezzocannone 16, 80134 Naples, Italy; concetta.avitabile@cnr.it; 3 Department of Pharmaceutical Sciences, University of Milan, Via Venezian 21, 20133 Milan, Italy; alessandra.romanelli@unimi.it; 4 BAT Center-Interuniversity Center for Studies on Bioinspired Agro-Environmental Technology, University of Naples Federico II, Via Università 100, Portici, 80055 Naples, Italy; 5 Task Force on Microbiome Studies, University of Naples Federico II, 80131 Naples, Italy

**Keywords:** crop protection, signaling peptide, plant defense, foliar application, hydroponics, antioxidant activity

## Abstract

Plant defense peptides are able to control immune barriers and represent a potential novel resource for crop protection. One of the best-characterized plant peptides is tomato Systemin (Sys) an octadecapeptide synthesized as part of a larger precursor protein. Upon pest attack, Sys interacts with a leucine-rich repeat receptor kinase, systemin receptor SYR, activating a complex intracellular signaling pathway that leads to the wound response. Here, we demonstrated, for the first time, that the direct delivery of the peptide to *Solanum melongena* and *Vitis vinifera* plants protects from the agent of Grey mould (*Botrytis cinerea*). The observed disease tolerance is associated with the increase of total soluble phenolic content, the activation of antioxidant enzymes, and the up-regulation of defense-related genes in plants treated with the peptide. Our results suggest that in treated plants, the biotic defense system is triggered by the Sys signaling pathway as a consequence of Sys interaction with a SYR-like receptor recently found in several plant species, including those under investigation. We propose that this biotechnological use of Sys, promoting defense responses against invaders, represents a useful tool to integrate into pest management programs for the development of novel strategies of crop protection.

## 1. Introduction

The success of modern agriculture relies in part on discovery and adoption of pesticides for pest control [1]. However, the onset of different concerns on the impact of pesticides on the environment, biodiversity, as well as on human health, pressed the introduction of more stringent pesticide registration procedures. Furthermore, the tendency in European Union policy is to encourage the development of eco-friendly and sustainable control strategies to protect crops reducing chemical inputs [2]. One of the main challenges facing the agricultural sector is to reduce the negative impact on soil, water, and the atmosphere.

Sustainable strategies for pest control have been applied to agricultural practices, such as biological control. This approach includes the use of beneficial microorganisms or bioactive compounds that bio-stimulate plant performance against pathogens by competing or by directly antagonizing them [3,4,5,6]. Some other alternative control strategies of plant diseases are based on the use of plant resistance inducers (PRIs, also called elicitors or plant defense/resistance activators), which offer the prospect of durable, broad-spectrum disease control [7]. PRIs can be chemical compounds [8] as well as biological stimulators [9] able to activate and/or prime plant defense responses by their exogenous application [10]. Depending on their nature, they either mimic plant downstream signaling molecules, such as phytohormone or derivates, or act as non-self molecules, classified as microbe/pathogen/herbivore-associated molecular patterns (MAMPs/PAMPs/HAMPs), or signals from damaged cells, generally referred to danger- or damage-associated molecular patterns (DAMPs) [11,12,13,14] or phytocytochines [15]. PRIs are recognized by plasma-membrane localized pattern recognition receptors (PRRs) to initiate signal transduction pathway [7]. One of the best characterized DAMP is systemin (Sys), an octadecapeptide synthesized as a part of a larger precursor protein, prosystemin (ProSys) [16,17]. Sys was isolated from tomato leaves and proved to be able to activate the octadecanoid pathway, which leads to the production of the plant hormone jasmonic acid (JA) and its derivatives, powerful activators of plant defense genes [18,19]. Transgenic tomato plants constitutively expressing *ProSys* proved to be resistant to insect herbivores and phytopathogenic fungi [20,21,22] and tolerant to moderate salt stress [23]. Homologs of the tomato *ProSys* gene have been identified only in some economically important species of Solanoideae subfamily, but other genetically distinct families of plant defense signal peptides have been described in several species [24,25,26,27,28,29,30].

Upon either pests or other environmental challenges cues, Sys interacts with a leucine-rich repeat receptor like-kinase (LRR-RLK), RLK SYSTEMIN RECEPTOR 1 (SYR1) and with lower affinity its homologous SYR2, triggering a complex intracellular signaling pathway that leads to the generation of early and late defense responses [31]. It was recently observed that although both SYR1 and SYR2 receptors are restricted to the species of Solanoideae subfamily (e.g., tomato, potato, eggplant, and pepper), other SYR-like genes are present in other plants species, including *Vitis vinifera* [31].

Sys perception at the cellular surface induces depolarization of the plasma membrane, mitogen-activated protein kinases (MAPKs), the opening of ion channels, with the consequent increase of intracellular Ca^2+^ concentration, and accumulation of reactive oxygen species (ROS) [32].

Since ROS participate in signaling events, they are highly reactive but also toxic to the cells. To control the level of ROS and protect cells under stress conditions, plants have developed a sophisticated ROS scavenging system that includes the activity of several enzymes such as catalase (CAT) and ascorbate peroxidase (APX) as well as non-enzymatic low molecular compounds such as phenolics compounds [33,34,35,36].

Eggplants (*Solanum melongena* L.) and grapevine (*Vitis vinifera* L.) are particularly susceptible to important fungal pathogens, among them *Botrytis cinerea*, the agent causing grey mold which diminish yield and depreciate quality throughout their entire biological cycle [37,38]. Phytochemicals are commonly used to prevent and reduce the damages of this pathogen infection, but pathogen strains with pesticide-resistance have been reported [39,40]. In an effort to protect crops from such a dangerous enemy and yet reduce the impact of chemicals on the environment, considerable interest has been focused on the identification of novel biotechnological tools that use elicitors to strengthen the endogenous defenses of plants. In this work, we demonstrated that the direct delivery of Sys to *Vitis vinifera* and *Solanum melongena* plants strongly reduces *B. cinerea* plant colonization.

## 2. Materials and Methods

### 2.1. Peptides

Two different purified peptides were assayed: Systemin (Sys) and its scrambled form (Scp) that does not activate the plant defense response in tomato. Peptides synthesis, purification, and stability are reported elsewhere [41].

### 2.2. Plant Materials and Growth Conditions

The eggplant variety used was “Violetta Lunga”. For this crop, two different growth systems were carried out: In soil and in hydroponic culture.

Seeds were germinated in Petri dishes on wet sterile paper and kept in the dark for three days in a growth chamber at 24 ± 1 °C and 60% relative humidity (RH). Upon roots emergence, for soil culture, eighteen plantlets were transferred to a polystyrene plateau with inert substrate S-type (Floragard, Oldenburg, Germany) in a growth chamber at 26 ± 1 °C and 60% RH with a photoperiod of 18/6 h light/dark. After two weeks, plants were transplanted in pots of 9 cm diameter with sterile soil mixture using the same growth conditions. For hydroponic culture, eighteen plantlets of 2 cm were transferred to hydroponic system and divided into three different plastic containers (5 L) supplemented with Mg(NO_3_)_2_·6H_2_O (384 mg/L), Ca(NO_3_)_2_·4H_2_O (812.9 mg/L), KNO_3_ (101.5 mg/L), K_2_SO_4_ (319.3 mg/L), KH_2_PO_4_ (204.8 mg/L), Hydromix (14.0 mg/L). Four weeks-old plants were used for biological and molecular investigations unless otherwise indicated.

Grapevine, cultivar “Cabernet Sauvignon” cuttings (rootstock genotype 101.14 CL. 759), were grown in a greenhouse in pots of 20 cm diameter until they developed six to eight leaves. The second and third youngest adult leaves from each cutting were used for biological and molecular investigations.

### 2.3. Plant Treatments with Peptides and Botrytis cinerea Assay

Intact leaves of eggplant and grapevine plants grown in soil were treated with 100 pM of Sys or Scp peptides in PBS buffer (phosphate buffer saline, 10 mM phosphates, 140 mM NaCl, 2.7 mM KCl, pH 7.4, Sigma-Aldrich, Milan, Italy) while to eggplants growing in hydroponics, peptides were added into a nutrient solution at the same final concentration. Control plants were similarly treated with PBS buffer.

Four weeks-old plants, leaf-treated or grown in hydroponics enriched with the Sys or Scp, were tested for resistance to the necrotrophic airborne pathogen, *B. cinerea*, as already reported [42]. The assay used five leaves per treatment from three different plants per each thesis. Control and treated leaves were placed on sponges soaked in sterile water and incubated in a growth chamber at 23 ± 1 °C under 16/8 h light/dark photoperiod and 90% RH as also described by [43,44].

Necrosis areas were measured at 1, 3, 5, and 7 days post inoculum (pi) with a digital caliber (Neiko 01407A, Neiko Tools, Taiwan, China).

### 2.4. In Vitro Antifungal Assay

The antifungal assay was carried out as already reported [45]. Briefly, a sterile 12-well plate was filled with potato dextrose broth (PDB 1/2) medium containing Sys and Scp peptides at the final concentration of 100 pM. A solution with *B. cinerea* spores was added to each well in order to reach a final concentration of 10^4^ spores/mL in each well, the plate was placed in a shaker and incubated for 24 h at 25 ± 1 °C. To assess the fungal growth, the value of optical density (OD) at a wavelength of 600 nm was measured in triplicate on a BioPhotometer Spectrophotometer UV/VIS (Eppendorf, Hamburg, Germany).

### 2.5. Gene Expression Analyses

Total RNA extraction, single-strand cDNA synthesis, and quantitative reverse transcription (RT)-PCR were performed as already reported [46]. Expression analysis of selected defense-related genes was monitored 3 h and 6 h after Sys foliar and hydroponic application, respectively. Gene expression analysis was carried out using two technical replicates for each of the three biological replicates. Relative quantification of gene expression was carried out using the comparative method with the 2^−ΔΔCt^ formula [47] where ΔCt = Ct target gene—Ct endogenous control and ΔΔCt = ΔCt sample—ΔCt calibrator. The housekeeping *APRT* (*adenine phosphoribosyl transferase*) and the *EF-1α* (*elongation factor-1α*) genes were the endogenous reference genes, respectively, for eggplant and grapevine plants, used for the normalization of the expression levels of the target genes. Primers and related genes under investigation are listed in Table S1.

### 2.6. Biochemical Analyses

Total phenolic content (TPC) and antioxidant enzyme activities were assessed spectrophotometrically in treated leaves of eggplant and grapevine plants collected at various time intervals: 1, 3, 6, and 24 h after peptides treatment using three technical replicates for each of the three biological replicates. Untreated leaves were used as control.

For the extraction of total soluble proteins, frozen leaf sample (0.1 g) was ground with 1 mL ice-cold 50 mM KHPO_4_ (pH 7.8) containing 0.1 mM EDTA. Homogenates were centrifuged at 14,000 rpm for 20 min at 4 °C.

Protein concentration was measured by the Bradford method using bovine serum albumin as a standard protein [48]. TPC was evaluated by using Folin–Ciocalteu colorimetric method as described before [49].

The catalase (CAT) activity was measured following the previously described protocols [50,51], monitoring the decrease in absorbance at 240 nm. Ascorbate peroxidase (APX) activity was analyzed by measuring the decrease in absorbance at 290 nm monitored according to the method previously described [52].

### 2.7. Statistical Analyses

For the evaluation of Sys effect on *B. cinerea* growth and infection, necrosis area differences between controls and Sys-treated or Scp-treated sample were compared and analyzed by one-way Analysis Of Variance (ANOVA) coupled with Tukey–Kramer Honestly Significant Difference (HSD) test. Differences in relative quantities of defense transcripts were analyzed by comparing ΔCt values for all the replicates of tests and controls using a two-tailed Student’s t-test. Moreover, the quantification of the amount of total phenolic content and the evaluation of the activities of antioxidant enzymes were analyzed by one-way ANOVA coupled with Tukey–Kramer multiple comparisons test. Error bars referring to standard error have been displayed.

## 3. Results

### 3.1. Systemin Exogenous Supply Reduces B. cinerea Colonization of Eggplant and Grapevine Leaves

**Figure 1:**
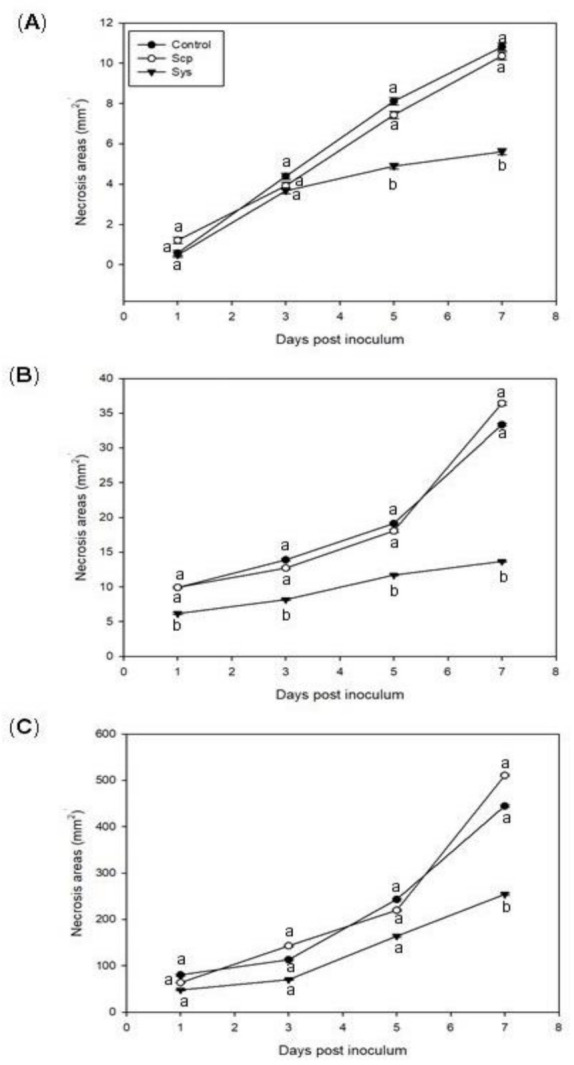
*Botrytis cinerea* necrosis area assay. Sys was applied to eggplant leaves (**A**) or added to hydroponic solution (**B**), while for the grapevine plants, only leaves were treated (**C**). Response to *B. cinerea* infection on leaves from plants treated with 100 pM Sys or Scp or Control (PBS 1X). The graph displays the average (±S. E., standard error) of the lesion size at 1, 3, 5, and 7 days post-inoculation. Letters indicate statistically significant differences (one-way Analysis of Variance, ANOVA, Tukey–Kramer Honestly Significant Differences (HSD) test with *p* < 0.05). Error bars indicate standard error.

The performance of Sys-treated eggplants and grapevine against *B. cinerea* was evaluated at 1, 3, 5, and 7 days post inoculum (pi). The assay was carried out using detached leaves harvested 6 h after peptides, Sys or Scp, application to intact plants [41,42]. Disease severity was quantified by measuring the necrotic leaf areas caused by fungal colonization. In eggplants, as shown in Figure 1, Sys significantly reduced the lesions since five days pi (Figure 1A), whereas in leaves deriving from hydroponic cultures, a reduction of the lesions was evident already 24 h pi (Figure 1B). No differences were observed for eggplants treated with buffer and Scp-peptide. Similarly, grapevine Sys-treated leaves displayed a marked reduction of *B. cinerea* induced lesions after seven days pi compared with the control ones (Figure 1C). Likewise to the previous experiment, no effect was detected in Scp or buffer treated leaves. These results demonstrate that the exogenous supply of Sys peptide to healthy plants reduced disease severity.

Moreover, in order to evaluate whether the reduction of *B. cinerea* necrosis area was due to a direct antimicrobial effect of the Sys peptide on the fungus, an in vitro assay to measure fungal growth in the presence of Sys and Scp peptides was carried out.

**Figure 2:**
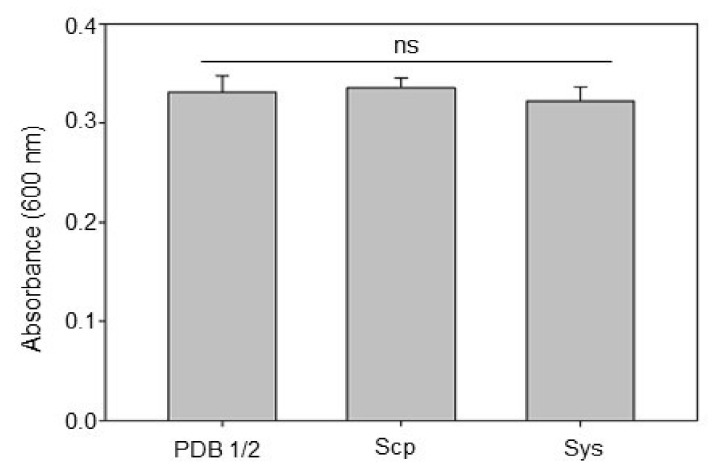
In vitro antifungal vitality assay. Each 12-well sterile plate was filled with 1 mL of PDB 1/2 medium containing the peptides at the final concentration of 100 pM, except for the broth sterility control wells. Thereafter, spores of *B. cinerea* were added to each well, and fungal growth was assessed 24 h after pathogen inoculation by evaluating the optical density (OD) of the medium at 600 nm. Letters indicate statistically significant differences (one-way ANOVA, Tukey–Kramer Honestly Significant Differences (HSD) test with *p* < 0.05; ns, not significant). Error bars indicate standard error.

As shown in Figure 2, Sys peptide did not directly impact fungus vitality. The growth of *B. cinerea*, monitored by measuring the absorbance at 600 nm, was similar in all three treatments. This result indicates that the observed reduction of *B. cinerea* plant colonization is determined by the induction of plant endogenous defenses upon Sys treatment.

### 3.2. Systemin Exogenous Supply Activated the Expression of S. melongena and V. vinifera Defense-Related Genes

In order to verify the ability of Sys-treatments to induce the expression of defense-related genes, we performed a qRT-PCR of selected genes for the two plant species. The genes analyzed were: *Allene Oxide Synthase* (*AOS*), *Wound-induced proteinase inhinbitor I* and *II* (*Pin I* and *Pin II*), *Pathogenesis-related protein 4* (*PR4*), *Dihydroflavonol 4-reductase* (*DFR*) and *Polyphenol oxidase* (*PPO*) for eggplants, the *basic-helix-loop-helix* (*bHLH*) *transcription factor* (*TF*) (*MYC2*), *AOS*, *Pin I*, *Pin II*, *PR4*, *Phenylalanine ammonia-lyase* (*PAL*) and *Flavonol synthase 5* (*FLS5*) for grapevine plants. The expression of the target genes was analyzed at two time intervals after treatment. Relative quantification of treated samples was referred to the mock-treated control (relative quantification, RQ = 1).

**Figure 3:**
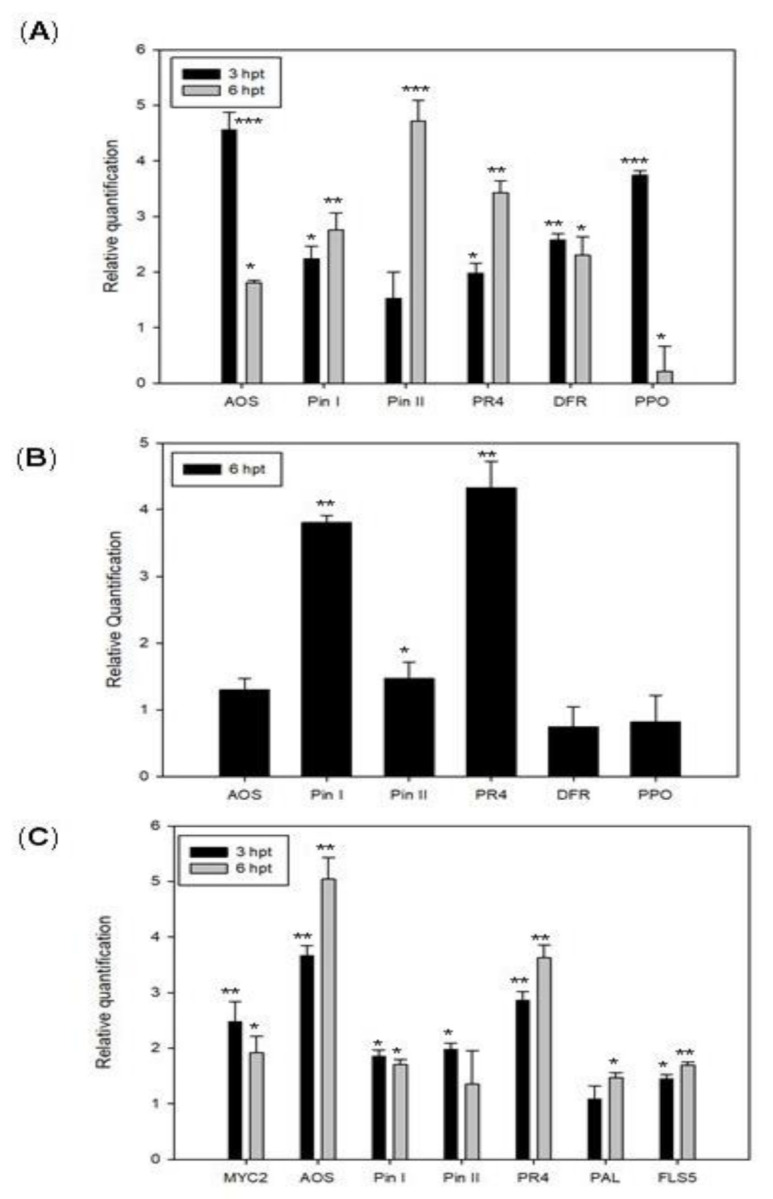
Expression analysis of defense-related genes following Sys treatments (100 pM) on eggplants and grapevine plants. Relative gene expression of defense-related genes by qRT-PCR in eggplants-treated leaves (**A**), in leaves of eggplants grown in a hydroponic system (**B**) and in grapevine-treated leaves (**C**). Quantities are relative to the calibrator control condition, mock-treated plants. Asterisks indicate data statistical significance (Student’s *t*-test, * *p* < 0.05, ** *p* < 0.01, *** *p* < 0.001). Error bars indicate standard error.

As shown in Figure 3A, in eggplants, a strong increase of *AOS* transcript was recorded 3 h after Sys application followed by a reduction of the transcript after 6 h from peptide application. Conversely, the expression profile of *Pin I* and *II* showed a gradual increase in their transcripts that reached the highest expression level 6 h after Sys treatment. Moreover, *PR4*, *DRF*, and *PPO* transcripts resulted significantly up-regulated (Figure 3A). We also monitored the expression of the same genes in leaves of eggplants grown in hydroponics enriched with the peptide. As shown in Figure 3B, *Pin I*, *Pin II*, and *PR4*, transcripts resulted significantly up-regulated after 6 h and no significant variation in transcript level was recorded for the other three genes.

Figure 3C shows the results of the gene expression analyses in treated leaves of grapevine plants. All the target transcripts resulted significantly up-regulated. Taken together, the results demonstrate that Sys, under two different delivery systems, is able to induce the transcription of defense-related genes in both plant species.

### 3.3. Systemin Increases the Production of Total Soluble Phenolic Content and Antioxidant Capacity in Treated Eggplant and Grapevine

**Figure 4:**
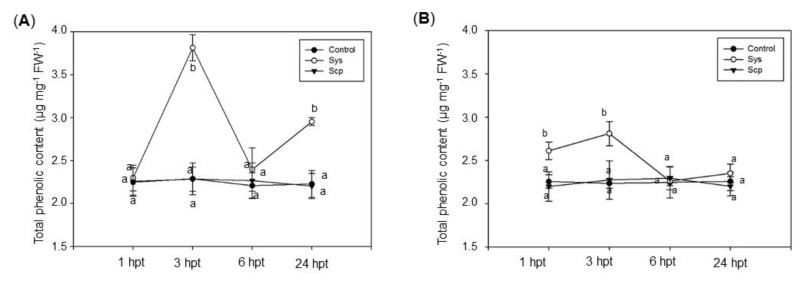
Total phenolic content (TPC) in eggplant (**A**) and grapevine (**B**) leaves treated with Sys. TPC was measured in control (PBS1X) and in treated leaves at 1, 3, 6, and 24 h after 100 pM Scp or Sys application. Letters indicate statistically significant differences (one-way ANOVA, Tukey test with *p* < 0.05). Error bars indicate standard error.

We quantified the amount of total phenolic content (TPC) and analyzed the activities of some key antioxidant enzymes that are responsible for rapid scavenging of ROS. Sys induced in treated plants a rapid antioxidant response, the TPC pool increased significantly by about 70% in eggplants (Figure 4A) 3 h after Sys application while the response of grapevine plants was more rapid with the increase of TPC after 1 h of roughly 16%. In addition, the TPC content in the treated plant species reached the highest content 3 h after Sys treatment (Figure 4, Table S2). On the contrary, as expected, the application of Scp peptide to the plants did not induce any TPC content variation (Figure 4, Table S2).

**Figure 5:**
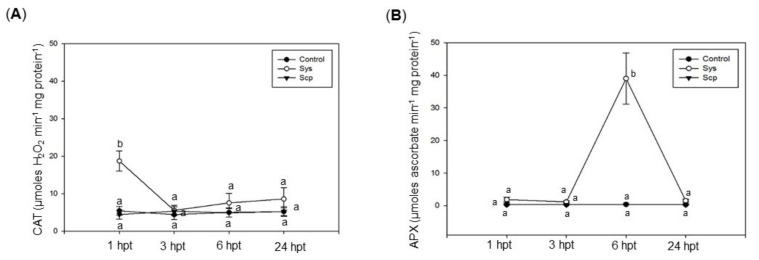
Catalase (CAT) (**A**) and ascorbate peroxidase (APX) (**B**) activity at various time intervals in eggplant leaves treated with Sys. CAT and APX activity was assessed in control leaves (PBS1X) and in treated leaves at 1, 3, 6, and 24 h after 100 pM Sys and Scp application. Letters indicate statistically significant differences (one-way ANOVA, Tukey test with *p* < 0.05). Error bars indicate standard error.

**Figure 6:**
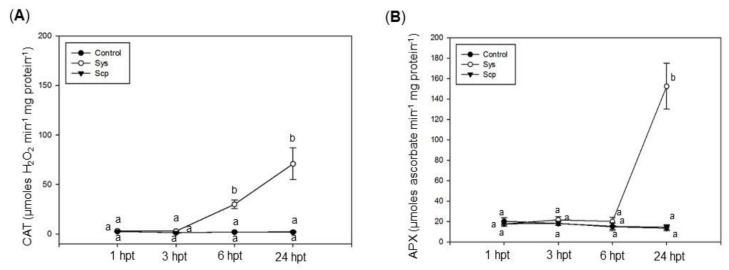
Catalase (CAT) (**A**) and ascorbate peroxidase (APX) (**B**) activity at various time intervals and grapevine leaves treated with Sys. CAT and APX activity was assessed in control leaves (PBS1X) and in treated leaves at 1, 3, 6, and 24 h after 100 pM Sys and Scp application. Letters indicate statistically significant differences (one-way ANOVA, Tukey test with *p* < 0.05). Error bars indicate standard error.

In addition to the investigation on the non-enzymatic components that regulate redox status, we monitored two enzymes that are included in the other arm of the antioxidant defense machinery. A significant increase in the activities of CAT and APX enzymes was observed in eggplant-treated leaves, respectively, of about four times and 100 times higher than control, 1 h and 6 h following Sys application, respectively (Figure 5A,B, Table S3). A different profile of CAT activity was observed in grapevine-treated leaves, which showed a steady increase after 3 h up to 40 times higher the control value 24 h post-treatment (Figure 6A, Table S4). In the same species, a significant increase in APX, about 11 times control value, was observed 24 h post-treatment (Figure 6B, Table S4). No significant variation in the activity of those enzymes was registered in leaves treated with Scp (Figure 5 and Figure 6, Tables S3 and S4).

## 4. Discussion

The development of safe and sustainable crop protection strategies is a challenging goal facing our society. This is increasingly pursued through bio-inspired research efforts, aiming to mimic natural mechanisms of pest suppression by exploiting biotechnological applications of biomolecules active in plant defense [53]. A promising control strategy is based on the application of elicitors to the plant that stimulate and/or potentiate plant defense responses affecting the fitness and behavior of herbivores and pathogens [42,54].

Among pathogenic plant agents, the necrotrophic fungus *B. cinerea* is a very dangerous fungus that infects many economically important crops, such as grapevine, strawberry, tomato, and eggplant. Grapevine is one of the major fruit crops in the world based on hectares cultivated with this crop and its economic value [55]. The species is particularly sensitive to various pathogenic fungi, including *B. cinerea* that causes significant losses in terms of production and quality. This pathogen is controlled by fungicide treatments, but pathogen strains with fungicide resistance have been reported [39]. Eggplant is one of the most important vegetable crops, especially for the Mediterranean basin, after potato (*Solanum tuberosum*) and tomato (*Solanum lycopersicum*) [56]. The plants are very susceptible to important fungal pathogens, including *B. cinerea*, throughout their entire biological cycle and the fungal control has been adversely affected by the development of fungicide resistance [40]. Therefore, the identification of novel biotechnological tools able to protect these crops from such a dangerous enemy is of great importance.

In this paper, we investigated the ability of tomato Sys to protect *S. melongena* and *V. vinifera* plants from *B. cinerea,* demonstrating, for the first time, that the exogenous supply of the peptide to intact healthy plants severely counteracted fungal growth. This is likely the consequence of the induction of plants defense-related genes that promote the accumulation of compounds active in plant defense [10,57]. Consequently, Sys-treated plants respond more effectively than controls when exposed to biotic stress. Both peptide delivery systems (leaf application or hydroponics uptake) proved to be very effective in conferring measurable protection against the necrotrophic fungus. The absence of inhibition of mycelium growth in the presence of Sys fully excluded that the peptide has a direct effect on the fungus. Therefore, the observed reduction of plant colonization is likely the consequence of the activation of plant endogenous defenses following Sys treatment. As a matter of fact, we observed the induction of a set of defense-related genes. *AOS*, a gene of the octadecanoid pathways, leads to the biosynthesis of JA that subsequently activate the late defense genes *PPO*, *Pin I*, and *Pin II*. Tomato *PPO* is induced by Sys and jasmonate, and it is involved in defense against pests [58,59]. In addition, *PPO* and *protease inhibitors* (*PIs*) are up-regulated by tobacco Sys as well as by the endogenous supply of a JA derived compound, the methyl jasmonate (MeJA) [60,61]. It has been demonstrated that PIs are very effective against *B. cinerea* both in vitro and in vivo: PIs isolated from young cabbage leaves were able to inhibit *B. cinerea* spore germination and germ tube elongation in vitro [62], whereas a strong inhibitory activity of a PIs mixture purified from tuber sprouts was observed against *B. cinerea* spore germination, germ tube elongation, and necrotic symptom development in vivo [63]. We also observed that the exogenous supply of Sys, under two different delivery systems, is able to induce the transcription of *PR4* genes in the two species. Pathogenesis-related proteins are a group of proteins involved in higher-plant responses to biotic stresses, whose expression is triggered by several pathogens, including fungi, bacteria, and viruses [64]. Many in vitro studies revealed that over-expression in various crops of *PR* genes (*PR2*, *PR3*, *PR4*, *PR5*, *PR12*), alone or in combination, leads to enhanced disease resistance against biotrophic and necrotrophic fungal phytopathogens [65]. Therefore, the disease reduction observed in our experimental plants is likely due, at least in part, to the increased level of protease inhibitors, polyphenol oxidase and PR4. Sys-treated eggplants showed an increased level of *DFR* transcript. *DFR*, together with *PAL*, *CHS*, *CHI* represents an essential component of the anthocyanin biosynthetic pathway. Developmental stages, diverse stresses, such as drought, temperature, wounding, and pathogen attack, are known to regulate anthocyanin biosynthesis. Previous studies showed that MeJA significantly induces anthocyanin accumulation through the up-regulation of genes encoding for anthocyanin biosynthetic enzymes, such as *DFR*, *LOX*, and *UF3GT* [66,67]. Sys-treated eggplants likely increase the MeJA production that may modulate the anthocyanin biosynthetic pathway [68].

Moreover, in grapevine, we observed that Sys application activated the phenylpropanoid pathway, as shown by the increased level of *PAL* transcript, and the induction of *MYC2* and *FLS5* genes. PAL, the first enzyme of the phenylpropanoid pathway, is involved in the biosynthesis of secondary metabolites, especially the production of phytoalexins and salicylic acid (SA) which were proposed to reduce the incidence of plant disease through antifungal activity and to stimulate plant defense responses, respectively [69,70]. It has also been shown that priming of *PAL1* is associated with responses to pathogen infection and wounding [71]. Interestingly, it was recently demonstrated that the exogenous application of MeJA in grapevine raises *PAL* gene expression and the consequent accumulation of several bioactive compounds (e.g., total phenolic and anthocyanin concentration) [72,73]. Therefore, in grapevine like in eggplant, Sys may induce an increase of MeJA that likely contribute to the accumulation of defense compounds. In addition, the up-regulation of *MYC2*, in Sys-treated grapevine plants, linked to the observed disease reduction, confirmed that this transcription factor is required for JA-mediated defense responses against the necrotrophic fungus *B. cinerea* [74].

Flavonols are the most abundant component of flavonoids, important secondary metabolites with a myriad of functions, including plant defense following pathogen attack, thanks to their antioxidant properties [75]. The increased level of *FLS5* transcripts registered in Sys-treated grapevine plants may favor the accumulation of these compounds that reduce disease severity following fungal infection.

Taken together, the most likely explanation of these results is the ability of Sys to bind SYR-like receptors or closely related genes recently identified in eggplants and grapevine plants, besides other plant species [31]. Following Sys-SYR interaction, the initiated signaling pathway leads to the systemic defense responses by the induction of JA synthesis that triggers the plant defense machine able to reduce the growth of with *B cinerea*.

It was previously shown that in tomato Sys causes very rapid changes in cellular redox homeostasis with the generation of excessive ROS [76,77], which may damage cell organelles. Since our data show that Sys is perceived by both eggplants and grapevine plants, in Sys treated plants, ROS likely increased and the plants reacted by activating the antioxidant defense machinery that boosted the TPC and the activity of CAT and APX enzymes, two key actors of the enzymatic H_2_O_2_ scavenging mechanism in plants [78].

Generally, in plants, the metabolism of H_2_O_2_ is controlled by several antioxidant scavenging enzymes, such as SOD, APX, and CAT [79,80]. The increased level of CAT and APX activities observed in treated plants of both species is likely functionally related to the cell requirement of a reduction of redox potential caused by Sys treatment. Similarly, the increased level of phenolic compounds may be linked to this function. In fact, they participate as antioxidants in the prevention of the plant from suffering molecular damage caused by microorganisms, insects, and herbivores [81]. In addition, it is worth noting that phenolic compounds play an important role in plant disease resistance responses representing an early defense plant reply to several biotic stresses [82]. As they are toxic to pathogens, their accumulation at the infection site can restrict pathogen development and the successive plant colonization or contrast infections by increasing the mechanical strength of the host cell wall [83]. Jasmonates (JAs), or their derivates, enhance the accumulation of phenolic compounds in different plant species contributing to the resistance against *B. cinerea* [84,85] and have a pivotal role in the reduction of H_2_O_2_ level by the enhancement of antioxidant enzymes activity in plant cells [86,87]. Sys-treated plants likely increase the JAs production that may modulate the activity of CAT and APX antioxidant enzymes in both plant species. Previous studies showed that the application of MeJA to in vitro cultures induced not only the expression of defense-related genes but also the antioxidant enzyme activity and the over-production of secondary metabolites [86]. Our results demonstrate the increase of both phenolic content and the antioxidative activity of CAT and APX enzymes likely determined by the activation of the JA pathways triggered by Sys treatment. In our experimental conditions, the increased level of TPC likely contributed to the observed reduction of damages on Sys treated leaves [84,88].

In conclusion, tomato systemin induces resistance against *B. cinerea,* indicating that the two species perceive the non-self-peptide and activate the defense and the antioxidant machineries. These results open a novel perspective on the use of plant peptides in crop protection. From an applied perspective, the exogenous delivery of plant signaling peptides integrated into pest management programs may offer a useful contribution to the reduction of chemical pesticide both in greenhouses and in the field.

## Data Availability

The data presented in this study are available in this article and Supplementary Material.

## Supplementary Materials

The following are available online at https://www.mdpi.com/2309-608X/7/1/15/s1. Table S1: Oligonucleotide sequence, gene symbol, accession number and plant species; Table S2: Effect of systemin peptide on total phenolic content at different times of leaf treatment in eggplant and grapevine plants; Table S3: Effect of systemin peptide on catalase (CAT) and ascorbate peroxidase (APX) activity at different times in eggplant treated leaves; Table S4: Effect of systemin peptide on catalase (CAT) and ascorbate peroxidase (APX) activity at different times in grapevine treated leaves.

## References

[1] Dayan F.E., Cantrell C.L., Duke S.O. (2009). Natural products in crop protection. Bioorg. Med. Chem..

[2] Fortunati E., Mazzaglia A., Balestra G.M. (2019). Sustainable control strategies for plant protection and food packaging sectors by natural substances and novel nanotechnological approaches. J. Sci. Food Agric..

[3] Ab Rahman S.F.S., Singh E., Pieterse C.M., Schenk P.M. (2018). Emerging microbial biocontrol strategies for plant pathogens. Plant Sci..

[4] Mansfield J., Genin S., Magori S., Citovsky V., Sriariyanum M., Ronald P., Dow M., Verdier V., Beer S.V., Machado M.A. (2012). Top 10 plant pathogenic bacteria in molecular plant pathology. Mol. Plant Pathol..

[5] Chiaiese P., Corrado G., Colla G., Kyriacou M.C., Rouphael Y. (2018). Renewable sources of plant biostimulation: Microalgae as a sustainable means to improve crop performance. Front. Plant Sci..

[6] Carillo P., Ciarmiello L.F., Woodrow P., Corrado G., Chiaiese P., Rouphael Y. (2020). Enhancing Sustainability by Improving Plant Salt Tolerance through Macro-and Micro-Algal Biostimulants. Biology.

[7] Marolleau B., Gaucher M., Heintz C., Degrave A., Warneys R., Orain G., Lemarquand A., Brisset M.-N. (2017). When a plant resistance inducer leaves the lab for the field: Integrating ASM into routine apple protection practices. Front. Plant Sci..

[8] Bektas Y., Eulgem T. (2015). Synthetic plant defense elicitors. Front. Plant Sci..

[9] Wiesel L., Newton A.C., Elliott I., Booty D., Gilroy E.M., Birch P.R., Hein I. (2014). Molecular effects of resistance elicitors from biological origin and their potential for crop protection. Front. Plant Sci..

[10] Goellner K., Conrath U. (2007). Priming: It’s all the world to induced disease resistance. Sustainable Disease Management in a European Context.

[11] Boller T., Felix G. (2009). A renaissance of elicitors: Perception of microbe-associated molecular patterns and danger signals by pattern-recognition receptors. Annu. Rev. Plant Biol..

[12] Ferrari S., Savatin D.V., Sicilia F., Gramegna G., Cervone F., De Lorenzo G. (2013). Oligogalacturonides: Plant damage-associated molecular patterns and regulators of growth and development. Front. Plant Sci..

[13] Albert M. (2013). Peptides as triggers of plant defence. J. Exp. Bot..

[14] Hou S., Liu Z., Wu D. (2019). Damage-Associated Molecular Pattern-Triggered Immunity in Plants. Front. Plant Sci..

[15] Gust A.A., Pruitt R., Nürnberger T. (2017). Sensing danger: Key to activating plant immunity. Trends Plant Sci..

[16] McGurl B., Pearce G., Orozco-Cardenas M., Ryan C.A. (1992). Structure, expression, and antisense inhibition of the systemin precursor gene. Science.

[17] McGurl B., Ryan C.A. (1992). The organization of the prosystemin gene. Plant Mol. Biol..

[18] Pearce G., Strydom D., Johnson S., Ryan C.A. (1991). A polypeptide from tomato leaves induces wound-inducible proteinase inhibitor proteins. Science.

[19] Ryan C.A., Pearce G. (2003). Systemins: A functionally defined family of peptide signals that regulate defensive genes in Solanaceae species. Proc. Natl. Acad. Sci. USA.

[20] Dıaz J., ten Have A., van Kan J.A. (2002). The role of ethylene and wound signaling in resistance of tomato to *Botrytis cinerea*. Plant Physiol..

[21] El Oirdi M., El Rahman T.A., Rigano L., El Hadrami A., Rodriguez M.C., Daayf F., Vojnov A., Bouarab K. (2011). *Botrytis cinerea* manipulates the antagonistic effects between immune pathways to promote disease development in tomato. Plant Cell..

[22] Coppola M., Corrado G., Coppola V., Cascone P., Martinelli R., Digilio M.C., Pennacchio F., Rao R. (2015). Prosystemin overexpression in tomato enhances resistance to different biotic stresses by activating genes of multiple signaling pathways. Plant Mol. Biol. Rep..

[23] Orsini F., Cascone P., De Pascale S., Barbieri G., Corrado G., Rao R., Maggio A. (2010). Systemin-dependent salinity tolerance in tomato: Evidence of specific convergence of abiotic and biotic stress responses. Physiol. Plant..

[24] Pearce G., Moura D.S., Stratmann J., Ryan C.A. (2001). Production of multiple plant hormones from a single polyprotein precursor. Nature.

[25] Schmelz E.A., Carroll M.J., LeClere S., Phipps S.M., Meredith J., Chourey P.S., Alborn H.T., Teal P.E. (2006). Fragments of ATP synthase mediate plant perception of insect attack. Proc. Natl. Acad. Sci. USA.

[26] Huffaker A., Pearce G., Ryan C.A. (2006). An endogenous peptide signal in *Arabidopsis* activates components of the innate immune response. Proc. Natl. Acad. Sci. USA.

[27] Huffaker A., Dafoe N.J., Schmelz E.A. (2011). ZmPep1, an ortholog of Arabidopsis elicitor peptide 1, regulates maize innate immunity and enhances disease resistance. Plant Physiol..

[28] Yamaguchi Y., Pearce G., Ryan C.A. (2006). The cell surface leucine-rich repeat receptor for AtPep1, an endogenous peptide elicitor in *Arabidopsis*, is functional in transgenic tobacco cells. Proc. Natl. Acad. Sci. USA.

[29] Yamaguchi Y., Barona G., Ryan C.A., Pearce G. (2011). GmPep914, an eight-amino acid peptide isolated from soybean leaves, activates defense-related genes. Plant Physiol..

[30] Pearce G. (2011). Systemin, hydroxyproline-rich systemin and the induction of protease inhibitors. Curr. Protein Pept. Sci..

[31] Wang L., Einig E., Almeida-Trapp M., Albert M., Fliegmann J., Mithöfer A., Kalbacher H., Felix G. (2018). The systemin receptor SYR1 enhances resistance of tomato against herbivorous insects. Nat. Plants.

[32] Zhang H., Zhang H., Lin J. (2020). Systemin-mediated long-distance systemic defense responses. New Phytol..

[33] Suzuki N., Koussevitzky S., Mittler R., Miller G. (2012). ROS and redox signalling in the response of plants to abiotic stress. Plant Cell Environ..

[34] Grene R. (2002). Oxidative stress and acclimation mechanisms in plants. Am. Soc. Plant Biol..

[35] Sofo A., Scopa A., Nuzzaci M., Vitti A. (2015). Ascorbate peroxidase and catalase activities and their genetic regulation in plants subjected to drought and salinity stresses. Int. J. Mol. Sci..

[36] Naikoo M.I., Dar M.I., Raghib F., Jaleel H., Ahmad B., Raina A., Khan F.A., Naushin F. (2019). Role and regulation of plants phenolics in abiotic stress tolerance: An overview. Plant Signaling Molecules.

[37] Buzatu M.A., Costache M., Hoza D., Șovărel G., Cristea S. (2018). The efficacy of different treatments for pathogens control on the eggplant crops in the field. Sci. Pap. Ser. B Hortic..

[38] Aziz A., Heyraud A., Lambert B. (2004). Oligogalacturonide signal transduction, induction of defense-related responses and protection of grapevine against *Botrytis cinerea*. Planta.

[39] Leroux P., Chapeland F., Desbrosses D., Gredt M. (1999). Patterns of cross-resistance to fungicides in *Botryotinia fuckeliana* (*Botrytis cinerea*) isolates from French vineyards. Crop Prot..

[40] Banno S., Yamashita K., Fukumori F., Okada K., Uekusa H., Takagaki M., Kimura M., Fujimura M. (2009). Characterization of QoI resistance in *Botrytis cinerea* and identification of two types of mitochondrial cytochrome b gene. Plant Pathol..

[41] Coppola M., Cascone P., Madonna V., Di Lelio I., Esposito F., Avitabile C., Romanelli A., Guerrieri E., Vitiello A., Pennacchio F. (2017). Plant-to-plant communication triggered by systemin primes anti-herbivore resistance in tomato. Sci. Rep..

[42] Coppola M., Lelio I.D., Romanelli A., Gualtieri L., Molisso D., Ruocco M., Avitabile C., Natale R., Cascone P., Guerrieri E. (2019). Tomato Plants Treated with Systemin Peptide Show Enhanced Levels of Direct and Indirect Defense Associated with Increased Expression of Defense-Related Genes. Plants.

[43] Rosero-Hernández E.D., Moraga J., Collado I.G., Echeverri F. (2019). Natural Compounds That Modulate the Development of the Fungus *Botrytis cinerea* and Protect *Solanum lycopersicum*. Plants.

[44] Gruau C., Trotel-Aziz P., Verhagen B., Villaume S., Rabenoelina F., Courteaux B., Clément C., Baillieul F., Aziz A. (2016). An Assay to Study *Botrytis cinerea*-infected Grapevine Leaves Primed with *Pseudomonas fluorescens*. Bio Protoc..

[45] Pastor-Fernández J., Gamir J., Pastor V., Sanchez-Bel P., Sanmartín N., Cerezo M., Flors V. (2020). Arabidopsis Plants Sense Non-self Peptides to Promote Resistance against *Plectosphaerella cucumerina*. Front. Plant Sci..

[46] Corrado G., Alagna F., Rocco M., Renzone G., Varricchio P., Coppola V., Coppola M., Garonna A., Baldoni L., Scaloni A. (2012). Molecular interactions between the olive and the fruit fly *Bactrocera oleae*. BMC Plant Biol..

[47] Livak K.J., Schmittgen T.D. (2001). Analysis of relative gene expression data using real-time quantitative PCR and the 2^−ΔΔCT^ method. Methods.

[48] Bradford M. (1970). A rapid and sensitive method for the quantification of microgram of protein utilizing the principle of protein dye banding. Ann. Biochem..

[49] Chiaiese P., Palomba F., Tatino F., Lanzillo C., Pinto G., Pollio A., Filippone E. (2011). Engineered tobacco and microalgae secreting the fungal laccase POXA1b reduce phenol content in olive oil mill wastewater. Enzyme Microb. Technol..

[50] Aebi H. (1984). [13] Catalase In Vitro. Methods in Enzymology.

[51] Haida Z., Hakiman M. (2019). A comprehensive review on the determination of enzymatic assay and nonenzymatic antioxidant activities. Food Sci. Nutr..

[52] Nakano Y., Asada K. (1981). Hydrogen peroxide is scavenged by ascorbate-specific peroxidase in spinach chloroplasts. Plant Cell Physiol..

[53] Pennacchio F., Giordana B., Rao R. (2012). Applications of parasitoid virus and venom research in agriculture. Parasitoid Viruses.

[54] Delaunois B., Farace G., Jeandet P., Clément C., Baillieul F., Dorey S., Cordelier S. (2014). Elicitors as alternative strategy to pesticides in grapevine? Current knowledge on their mode of action from controlled conditions to vineyard. Environ. Sci. Pollut. Res..

[55] Armijo G., Schlechter R., Agurto M., Muñoz D., Nuñez C., Arce-Johnson P. (2016). Grapevine pathogenic microorganisms: Understanding infection strategies and host response scenarios. Front. Plant Sci..

[56] Rotino G.L., Sala T., Toppino L. (2014). Eggplant. Alien Gene Transfer in Crop Plants.

[57] Conrath U., Beckers G.J., Flors V., García-Agustín P., Jakab G., Mauch F., Newman M.-A., Pieterse C.M., Poinssot B., Pozo M.J. (2006). Priming: Getting ready for battle. Mol. Plant Microbe Interact..

[58] Constabel C.P., Barbehenn R. (2008). Defensive roles of polyphenol oxidase in plants. Induced Plant Resistance to Herbivory.

[59] Mayer A.M. (2006). Polyphenol oxidases in plants and fungi: Going places? A review. Phytochemistry.

[60] Constabel C.P., Ryan C.A. (1998). A survey of wound-and methyl jasmonate-induced leaf polyphenol oxidase in crop plants. Phyt Chem..

[61] Ren F., Lu Y.-T. (2006). Overexpression of tobacco hydroxyproline-rich glycopeptide systemin precursor A gene in transgenic tobacco enhances resistance against *Helicoverpa armigera* larvae. Plant Sci..

[62] Lorito M., Broadway R., Hayes C., Woo S., Noviello C., Williams D., Harman G., Grings E., Adams D., Short R. (1994). Proteinase inhibitors from plants as a novel class of fungicides. Mol. Plant Microbe Interact..

[63] Hermosa M., Turra D., Fogliano V., Monte E., Lorito M. (2006). Identification and characterization of potato protease inhibitors able to inhibit pathogenicity and growth of *Botrytis cinerea*. Physiol. Mol. Plant Pathol..

[64] Dai L., Wang D., Xie X., Zhang C., Wang X., Xu Y., Wang Y., Zhang J. (2016). The Novel Gene VpPR4-1 from Vitis pseudoreticulata Increases Powdery Mildew Resistance in Transgenic *Vitis vinifera* L.. Front. Plant Sci..

[65] Ali S., Ganai B.A., Kamili A.N., Bhat A.A., Mir Z.A., Bhat J.A., Tyagi A., Islam S.T., Mushtaq M., Yadav P. (2018). Pathogenesis-related proteins and peptides as promising tools for engineering plants with multiple stress tolerance. Microbiol. Res..

[66] Loreti E., Povero G., Novi G., Solfanelli C., Alpi A., Perata P. (2008). Gibberellins, jasmonate and abscisic acid modulate the sucrose-induced expression of anthocyanin biosynthetic genes in Arabidopsis. New Phytol..

[67] Shan X., Zhang Y., Peng W., Wang Z., Xie D. (2009). Molecular mechanism for jasmonate-induction of anthocyanin accumulation in Arabidopsis. J. Exp. Bot..

[68] Belwal T., Singh G., Jeandet P., Pandey A., Giri L., Ramola S., Bhatt I.D., Venskutonis P.R., Georgiev M.I., Clément C. (2020). Anthocyanins, multi-functional natural products of industrial relevance: Recent biotechnological advances. Biotechnol. Adv..

[69] Trotel-Aziz P., Couderchet M., Vernet G., Aziz A. (2006). Chitosan stimulates defense reactions in grapevine leaves and inhibits development of *Botrytis cinerea*. Eur. J. Plant Pathol..

[70] Saigne-Soulard C., Abdelli-Belhadj A., Télef-Micouleau M., Bouscaut J.m., Cluzet S., Corio-Costet M.-F., Mérillon J.-M. (2015). Oligosaccharides from *Botrytis cinerea* and elicitation of grapevine defense. Polysacch. Bioactivity Biotechnol..

[71] Kohler A., Schwindling S., Conrath U. (2002). Benzothiadiazole-induced priming for potentiated responses to pathogen infection, wounding, and infiltration of water into leaves requires the NPR1/NIM1 gene in *Arabidopsis*. Plant Physiol..

[72] Hanaka A., Nurzyńska-Wierdak R. (2019). Methyl jasmonate—A multifunctional molecule throughout the whole plant life. Acta Sci. Polonorum. Hortorum Cultus..

[73] García-Pastor M.E., Serrano M., Guillén F., Castillo S., Martínez-Romero D., Valero D., Zapata P.J. (2019). Methyl jasmonate effects on table grape ripening, vine yield, berry quality and bioactive compounds depend on applied concentration. Sci. Hortic..

[74] Du M., Zhao J., Tzeng D.T., Liu Y., Deng L., Yang T., Zhai Q., Wu F., Huang Z., Zhou M. (2017). MYC2 orchestrates a hierarchical transcriptional cascade that regulates jasmonate-mediated plant immunity in tomato. Plant Cell..

[75] Lu Y., Chen Q., Bu Y., Luo R., Hao S., Zhang J., Tian J., Yao Y. (2017). Flavonoid accumulation plays an important role in the rust resistance of Malus plant leaves. Front. Plant Sci..

[76] Orozco-Cardenas M., Ryan C.A. (1999). Hydrogen peroxide is generated systemically in plant leaves by wounding and systemin via the octadecanoid pathway. Proc. Natl. Acad. Sci. USA.

[77] Orozco-Cárdenas M.L., Narváez-Vásquez J., Ryan C.A. (2001). Hydrogen peroxide acts as a second messenger for the induction of defense genes in tomato plants in response to wounding, systemin, and methyl jasmonate. Plant Cell..

[78] Apel K., Hirt H. (2004). Reactive oxygen species: Metabolism, oxidative stress, and signal transduction. Annu. Rev. Plant Biol..

[79] Mittler R. (2002). Oxidative stress, antioxidants and stress tolerance. Trends Plant Sci..

[80] Mittler R., Vanderauwera S., Gollery M., Van Breusegem F. (2004). Reactive oxygen gene network of plants. Trends Plant Sci..

[81] Hassanpour S., MaheriSis N., Eshratkhah B. (2011). Plants and secondary metabolites (Tannins): A Review. Int. J. For. Soil Eros..

[82] Sarabandi M., Farokhzad A., Mandoulakani B.A., Ghasemzadeh R. (2019). Biochemical and gene expression responses of two Iranian grape cultivars to foliar application of methyl jasmonate under boron toxicity conditions. Sci. Hortic..

[83] Miedes E., Vanholme R., Boerjan W., Molina A. (2014). The role of the secondary cell wall in plant resistance to pathogens. Front. Plant Sci..

[84] Wang H., Kou X., Wu C., Fan G., Li T. (2020). Methyl jasmonate induces the resistance of postharvest blueberry against gray mold caused by *Botrytis cinerea*. J. Sci. Food Agric..

[85] Min D., Li F., Cui X., Zhou J., Li J., Ai W., Shu P., Zhang X., Li X., Meng D. (2020). SlMYC2 are required for methyl jasmonate-induced tomato fruit resistance to *Botrytis cinerea*. Food Chem..

[86] Ho T.-T., Murthy H.N., Park S.-Y. (2020). Methyl Jasmonate Induced Oxidative Stress and Accumulation of Secondary Metabolites in Plant Cell and Organ Cultures. Int. J. Mol. Sci..

[87] Norastehnia A., Nojavan-Asghari M. (2006). Effect of methyl jasmonate on the enzymatic antioxidant defense system in maize seedling subjected to paraquat. Asian J. Plant Sci..

[88] Li S., Xu Y., Bi Y., Zhang B., Shen S., Jiang T., Zheng X. (2019). Melatonin treatment inhibits gray mold and induces disease resistance in cherry tomato fruit during postharvest. Postharvest Biol. Technol..

