# Supplementary material for: Colonization of Solanum melongena and Vitis vinifera Plants by Botrytis cinerea Is Strongly Reduced by the Exogenous Application of Tomato Systemin
Source: J Fungi (Basel). 2020 Dec 29;7(1):15. doi: 10.3390/jof7010015 (PMC7824362; doi:10.3390/jof7010015)
Supplement: Supplementary file 1 [file jof-07-00015-s001.zip › Supplementary Tables/Supplementary Table S1.docx]

**Table S1:** Oligonucleotide sequence, gene symbol, accession number and plant species

| **Primer** | **Sequence (5’-3’)** | **Gene symbol** | **Accession number** | **Plant species** |
| --- | --- | --- | --- | --- |
| APRT SM Fw | TGCATGTAGGTGCTGTGCAAG | *APRT* | FS056270.1 | *S. melongena* |
| APRT SM Rv | ACGCTCAAGAAGCCTAATCGC |  |  |  |
| AOS SM Fw | AGGCGAACTACTCTACGGGT | *AOS* | AF230371 | *S. melongena* |
| AOS SM Rv | TCTGTTTCGTGCCCATTCGA |  |  |  |
| PIN I SM Fw | CGGTTCTCCTGTCACTGCTG | *Pin I* | K03290 | *S. melongena* |
| PIN I SM Rv | GGCATTGATACACCAACACCC |  |  |  |
| PIN II SM Fw | AACCCTAACACTTGCCCTCT | *Pin II* | K03291 | *S. melongena* |
| PIN II SM Rv | GTAGCAACCCTTGTACCCCG |  |  |  |
| PR4 SM Fw | CGCTACTTGGGATGCTGACA | *PR4* | JX030397.1 | *S. melongena* |
| PR4 SM Rv | GCTTGTGTCCCTGTTCCTGT |  |  |  |
| DFR SM Fw | AGGACCCTGAGAATGGAGTAA | *DFR* | F074352.1 | *S. melongena* |
| DFR SM Rv | TCAAGAGTTCCAGCAGATGAAG |  |  |  |
| PPO SM Fw | AATGTACCGTCAATGGTAACTAA | *PPO* | 251851952 | *S. melongena* |
| PPO SM Rv | GTATGACGTCGAACCATTAGG |  |  |  |
| EF-1 Vitis Fw | GAACTGGGTGCTTGATAGGC | *EF1α* | GU585871.1 | *V. vinifera* |
| EF-1 Vitis Rv | AACCAAATATCCGGAGTAAAAGA |  |  |  |
| AOS Vitis Fw | GTCTCCCTTTCATCGGTCCC | *AOS* | FN595227 | *V. vinifera* |
| AOS Vitis Rv | TGACTGGTGTTTCTGGGCTC |  |  |  |
| MYC2 Vitis Fw | GGACGGAGGAGGATAAAGCG | *MYC2* | EF636725.2 | *V. vinifera* |
| MYC2 Vitis Rv | CATCACTCCCCACTGCCATT |  |  |  |
| PR4 Vitis Fw | GTGGTGGTGTTGCTGTCTCT | *PR4* | JN977472.1 | *V. vinifera* |
| PR4 Vitis Rv | CTCACTGCGTTCAAGTCCCA |  |  |  |
| PAL Vitis Fw | TCTGGTGGAAGGAATCCAAG | *PAL* | KU162973.1 | *V. vinifera* |
| PAL Vitis Rv | CAAAGTGCCACCAGGTAGGT |  |  |  |
| FLS5 Vitis Fw | GCATGTCATGGGCTGTGTTT | *FLS5* | AB213566.1 | *V. vinifera* |
| FLS5 Vitis Rv | ACTTGGCAGGGTTTGGTTCA |  |  |  |
| PIN I Vitis Fw | TTTCCTATTCCTGACCGCCG | *Pin I* | FN596025 | *V. vinifera* |
| PIN I Vitis Rv | ATGTTCTTGATCGGACGCCA |  |  |  |
| PIN II Vitis Fw | GCCTTGCTCGATTTGCTGTT | *Pin II* | FN596254 | *V. vinifera* |
| PIN II Vitis Rv | CTGCTCTTCTGCCTTCACCA |  |  |  |
