# Supplementary material for: Colonization of Solanum melongena and Vitis vinifera Plants by Botrytis cinerea Is Strongly Reduced by the Exogenous Application of Tomato Systemin
Source: J Fungi (Basel). 2020 Dec 29;7(1):15. doi: 10.3390/jof7010015 (PMC7824362; doi:10.3390/jof7010015)
Supplement: Supplementary file 1 [file jof-07-00015-s001.zip › Supplementary Tables/Supplementary Table S2.docx]

**Supplementary Table S2.** Effect of systemin peptide on total phenolic content at different times of leaf treatment in eggplant and grapevine plants

| **Species** | **Treatment** | **Total phenolic content (µg mg^-1^ FW^-1^)** | | | |
| --- | --- | --- | --- | --- | --- |
|  |  | **1 hpt** | **3 hpt** | **6 hpt** | **24 hpt** |
| *Solanum melongena* | Control | 2.25 ± 0.16a | 2.28 ± 0.18a | 2.21 ± 0.15a | 2.22 ± 0.15a |
|  | Sys | 2.30 ± 1.15a | 3.81 ± 0.15b | 2.39 ± 0.25a | 2.95 ± 0.05b |
|  | Scp | 2.26 ± 0.15a | 2.27 ± 0.14a | 2.26 ± 0.20a | 2.20 ± 0.14a |
| *Vitis vinifera* | Control | 2.25 ± 0.08a | 2.23 ± 0.06a | 2.24 ± 0.18a | 2.26 ± 0.11a |
|  | Sys | 2.61 ± 0.10b | 2.81 ± 0.14b | 2.26 ± 0.05a | 2.35 ± 0.10a |
|  | Scp | 2.19 ± 0.18a | 2.27 ± 0.22a | 2.29 ± 0.13a | 2.20 ± 0.11a |

Values are reported as the average of three independent measurements ± standard error (± S. E.). Different letters indicate significant differences according to Tukey multiple-range test (*P* < 0.05).
