# Supplementary material for: Colonization of Solanum melongena and Vitis vinifera Plants by Botrytis cinerea Is Strongly Reduced by the Exogenous Application of Tomato Systemin
Source: J Fungi (Basel). 2020 Dec 29;7(1):15. doi: 10.3390/jof7010015 (PMC7824362; doi:10.3390/jof7010015)
Supplement: Supplementary file 1 [file jof-07-00015-s001.zip › Supplementary Tables/Supplementary Table S3.docx]

**Supplementary Table S3.** Effect of systemin peptide on catalase (CAT) and ascorbate peroxidase (APX) activity at different times in eggplant treated leaves

| **Treatment** | **CAT (µmoles H_2_O_2_ min^-1^ mg protein^-1^)** | | | | **APX (µmoles ascorbate min^-1^ mg protein^-1^)** | | | |
| --- | --- | --- | --- | --- | --- | --- | --- | --- |
|  | **1 hpt** | **3 hpt** | **6 hpt** | **24 hpt** | **1 hpt** | **3 hpt** | **6 hpt** | **24 hpt** |
| **Control** | 5.41 ± 1.15a | 4.41 ± 1.35a | 5.01 ± 1.25a | 5.21 ± 1.25a | 0.33 ± 0.03a | 0.35 ± 0.04a | 0.39 ± 0.05a | 0.31 ± 0.02a |
| **Sys** | 18.71 ± 2.69b | 5.54 ±1.37a | 7.55 ± 2.56a | 8.49 ± 3.03a | 1.87 ± 0.74a | 1.15 ± 0.32a | 39 ± 7.80b | 1.51 ± 0.43a |
| **Scp** | 4.40 ± 1.21a | 5.34 ±1.17a | 4.89 ± 1.16a | 5.20 ± 1.03a | 0.29 ± 0.02a | 0.28 ± 0.04a | 0.34 ± 0.03a | 0.28 ± 0.03a |

Values are reported as the average of three independent measurements ± standard error (± S. E.). Different letters indicate significant differences according to Tukey multiple-range test (*P* < 0.05).
