# Supplementary material for: Colonization of Solanum melongena and Vitis vinifera Plants by Botrytis cinerea Is Strongly Reduced by the Exogenous Application of Tomato Systemin
Source: J Fungi (Basel). 2020 Dec 29;7(1):15. doi: 10.3390/jof7010015 (PMC7824362; doi:10.3390/jof7010015)
Supplement: Supplementary file 1 [file jof-07-00015-s001.zip › Supplementary Tables/Supplementary Table S4.docx]

**Supplementary Table S4.** Effect of systemin peptide on catalase (CAT) and ascorbate peroxidase (APX) activity at different times in grapevine treated leaves.

| **Treatment** | **CAT (µmoles H_2_O_2_ min^-1^ mg protein^-1^)** | | | | **APX (µmoles ascorbate min^-1^ mg protein^-1^)** | | | |
| --- | --- | --- | --- | --- | --- | --- | --- | --- |
|  | **1 hpt** | **3 hpt** | **6 hpt** | **24 hpt** | **1 hpt** | **3 hpt** | **6 hpt** | **24 hpt** |
| **Control** | 2.40 ± 0.47a | 1.40 ± 0.41a | 1.90 ± 0.25a | 1.80 ± 0.44a | 20.13 ± 3.45a | 18.11 ± 2.00a | 15.31 ± 2.95a | 14.41 ± 2.50a |
| **Sys** | 3.07 ± 0.35a | 2.13 ± 0.48a | 29.93 ± 4.31b | 70.82 ± 16.06b | 17.59 ± 2.32a | 21.62 ± 3.41a | 18.08 ± 3.98a | 152.4 ± 22.45b |
| **Scp** | 2.67 ± 0.51a | 1.18 ± 0.52a | 1.93 ± 0.28a | 2.13 ± 0.59a | 17.81 ± 2.21a | 18.92 ± 2.09a | 14.8 ± 2.86a | 13.49 ± 2.64a |

Values are reported as the average of three independent measurements ± standard error (± S. E.). Different letters indicate significant differences according to Tukey multiple-range test (*P* < 0.05).
